# Effectiveness of a 16-month multi-component and environmental school-based intervention for recovery of poor income overweight/obese children and adolescents: study protocol of the health multipliers program

**DOI:** 10.1186/s12889-017-4715-8

**Published:** 2017-09-15

**Authors:** Pollyanna Fernandes Patriota, Andrea Rocha Filgueiras, Viviane Belucci Pires de Almeida, Guilherme Aparecido Costa Alexmovitz, Carlos Eduardo da Silva, Vivian Fortuna Feres de Carvalho, Natália Carvalho, Maria Paula de Albuquerque, Semiramis Martins Alvares Domene, Wagner Luiz do Prado, Gustavo Enrique Salazar Torres, Ana Paula Reis de Oliveira, Ricardo Sesso, Ana Lydia Sawaya

**Affiliations:** 1 Department of Physiology, Federal University of São Paulo, UNIFESP, Rua Botucatu, 862, 2° andar, Vila Clementino. CEP, São Paulo, SP 04023062 Brazil; 2 Department of Public Health and Collective Health, Health and Society Institute, Federal University of São Paulo, UNIFESP, Santos, SP Brazil; 3 Department of Pediatrics, Federal University of São Paulo, UNIFESP, São Paulo, SP Brazil; 4 Center for Recovery and Nutritional Education – CREN, São Paulo, SP Brazil; 5 Department of Public Health and Collective Health, Health and Society Institute, UNIFESP, Santos, SP Brazil; 6 Department of Human Movement Sciences, UNIFESP, Santos, SP Brazil; 7 Department of Computer Science, Institute of Mathematics and Statistics, University of São Paulo, USP, São Paulo, SP Brazil; 8 Paulista School of Medicine, Federal University of São Paulo, UNIFESP, São Paulo, SP Brazil; 9 Department of Nephrology, Federal University of São Paulo, UNIFESP, São Paulo, SP Brazil

**Keywords:** Obesity, Nutrition education, Weight reduction programs, Clinical trials, Study protocol

## Abstract

**Background:**

Excess of weight is a serious public health concern in almost all countries, afflicting people of different ages and socioeconomic backgrounds. Studies have indicated the need for developing treatment strategies that intervene directly in the obesogenic environment. This study aims to evaluate the effectiveness of a multi-component and environmental school-based intervention, lasting 16 months, on the recovery of the nutritional status of low-income children and adolescents with overweight/ obesity.

**Methods/study design:**

The study was conducted by the Center for Recovery and Nutritional Education (CREN) in São Paulo, Brazil. Two schools located in poor neighborhoods were selected for the intervention, between March 2016 and June 2017. The participants were all students aged 8 to 12 years from the two participating schools. At the beginning of the intervention, anthropometric measurements were carried out to assess the nutritional status of the students. For convenience, students from one of the schools were considered as the control group, while those from the other school formed the experimental group. The intervention in the experimental group (*n* = 438) consists of the following weekly activities at school: psychological counseling in groups, theoretical/practical nutrition workshops, and supervised physical education classes. In addition, theoretical and practical educational activities are held regularly for parents, teachers, and cooks. Students with excess of weight (≥1 body mass index [BMI] –for-age Z score, *n* = 138) received clinical and nutritional care periodically at the outpatient care at CREN. Students enrolled in the control group (*n* = 353) participated in psychological counseling groups and theoretical/practical nutrition workshops for 6 months held in the school environment to provide motivation to entire classrooms. In the following 10 months, students with excess of weight from the control group (*n* = 125) were invited to attend the routine outpatient care at CREN.

**Discussion:**

This study is the first to assess the effectiveness of a multi-component and environmental school-based intervention for the recovery of low-income, overweight/obese children and adolescents. If positive, the results demonstrate the feasibility for the recovery of excess of weight in populations of similar conditions and age.

**Trial registration:**

Brazilian Registry of Clinical Trials - ReBEC Primary Id Number RBR-9t2jr8. Registration Date: Nov. 30, 2016. Retrospectively registered.

**Protocol version:** 3.

## Background

The World Health Organization (WHO) estimated 155 million or one in 10 school-age (5–17 years old) children worldwide to be either overweight or obese [1, 2]. The prevalence of excess of weight has markedly increased, not only in rich countries, but also in developing countries [3]. It is estimated that by 2025, Latin American countries with large populations, such as Brazil, Argentina, Chile, Peru, and Bolivia, could account for more than 75 million overweight children in the absence of effective intervention [4] .

The Brazilian Family Budget Survey shows that in children the prevalence of overweight and obesity among children is around 36% and 16%, respectively [5]. In adolescence, the prevalence of overweight and obesity is 20.5% and 5.9%, respectively. In large urban centers of the Southeast region, the prevalence of overweight is 38.8%. Among boys aged 10 to 19 years, the prevalence of overweight increased from 3.7% (1974–75) to 21.7% (2008–09). Among girls, the growth of excess weight was from 7.6% to 19.4% in the same age group. Obesity trends increased from 0.4% to 5.9% among boys and from 0.7% to 4% among girls. This situation reflects the nutritional transition scenario observed in Brazil, with a decline in the prevalence of childhood undernutrition and an increase in overweight at alarming levels across all age groups [5].

It is well known that weight gain is related to high consumption of low-nutrient products rich in sugars, fat, and salt (such as snacks and fast foods); routine consumption of sugary drinks; and insufficient physical activity [6–8]. From the nutritional perspective, it is recognized that personal eating preferences, purchasing decisions, and eating behaviors are influenced by price, marketing, availability, and accessibility. In turn, these factors are influenced by policies and regulations for agriculture and trade in food and beverages [9].

Since obesity is a multifactorial disease, effective interventions should have an environmental approach aimed at bringing about behavioral changes that impact on the obesogenic lifestyle [2, 10]. Low-income populations are the most affected, with less access to health care, and therefore, they are more likely to develop comorbidities associated with obesity [2, 5, 9]. Considering that behavioral change requires a sustained commitment by patients and families, motivation is the most important and most challenging aspect of obesity management [10]. A growing number of studies has shown that the motivational aspect is a predictor of success in the treatment of obesity, especially when it involves family members [11, 12].

Many children who have excess weight suffer from significant psychological problems, including anxiety, depression, attention-deficit hyperactivity, bullying, as well as other emotional and eating disorders [13, 14]. In addition, excess weight carries a social stigma that adversely affects children as well as their families [17]. For this reason, environmental interventions that include all children of a classroom, for example, can have better long-term benefits. This approach may facilitate youth leadership towards better health practices and reduce stigmatization of those with excess of weight [15].

Childhood excess of weight is a complex disease. This complexity makes it challenging to design interventions and test different treatment approaches at different settings. Traditionally, most treatments are administered exclusively in healthcare settings and do not offer a multi-component approach encompassing long-term educational and environmental interventions. For this reason, a treatment that includes a school-based intervention can offer a broader approach to changing feeding habits by including motivational groups during school classes, as well as nutrition education groups and supervised physical education classes. In addition, although recent reviews conclude that many interventions can induce weight loss in children and adolescents, effective and sustainable intervention protocols that allow the recovery of childhood excess of weight have yet to be identified [2], especially in low-income settings.

## Methods

### Study design

This is a controlled, prospective trial aimed at investigating the effectiveness of a 16-month, multi-component, and environmental school-based intervention for recovery of poor income overweight/obese students in which subjects are not randomly allocated. This study design was chosen since evidence increasingly has shown that randomized control trials (RTC) are not the most suited models to test the complex and chronic care required to treat children and adolescents with excess weight [15]. Moreover, the present design does not allow randomization of control and experimental groups, since the intervention involves the entire school environment.

### Study population

Two schools were chosen for this study among the three existing schools of the Southeast area of the city of São Paulo, Brazil. The schools have similar teaching schedules, are located in poor neighborhoods, and have students from equivalent socio-economic backgrounds. The selection criterion was their geographical location or proximity to the Center for Recovery and Nutritional Education (CREN), from where the Program is being managed. In all, 1003 children of the defined age group were enrolled in the two schools. After the first analysis, it was verified that 213 children did not meet the inclusion criteria, reducing the number of eligible participants to 791. The study included all the students between 8 to 12 years of age (*n* = 791) enrolled in the two schools. One school was chosen as the experimental group (*n* = 438), and the other was the control group (*n* = 353).

### Sample size calculation

A power analysis was conducted based on the changes in standardized BMI. Assuming an alpha error of 0.05, power of 0.80, and mean difference of the delta (post-intervention vs. baseline measurement) of standardized BMI of 0.12 relative to the control group, with a standard error of 0.30, the estimated number of participants need to be studied per group is 98 (total = 196) [16]. The estimated difference of 0.12 in the standardized BMI was based on previous intervention studies in obese children [17].

### Recruitment procedures

Discussions were held with the respective school principals to introduce the project, and after obtaining their formal approval, the intervention began. Parents/guardians were then invited to participate in the study through print communications and telephone calls and asked to attend to a meeting where the objectives and the research protocol were explained. On this occasion, they attended a lecture to raise awareness about the problem of excess weight and its impact on child health. After the lecture, parents and children willing to participate in the program were asked to sign the informed consent forms. The study protocol was approved by the Research Ethics Committee of Human Beings of the Federal University of São Paulo (CAAE: 34,304,714.40000.5505).

### Description of the intervention

The intervention aims at addressing all the possible actors involved in the obesogenic environment, such as family and school. The research team consists of a psychologist, four dietitians, a doctor, and a physical educator.

#### Experimental group

**Fig. 1:**
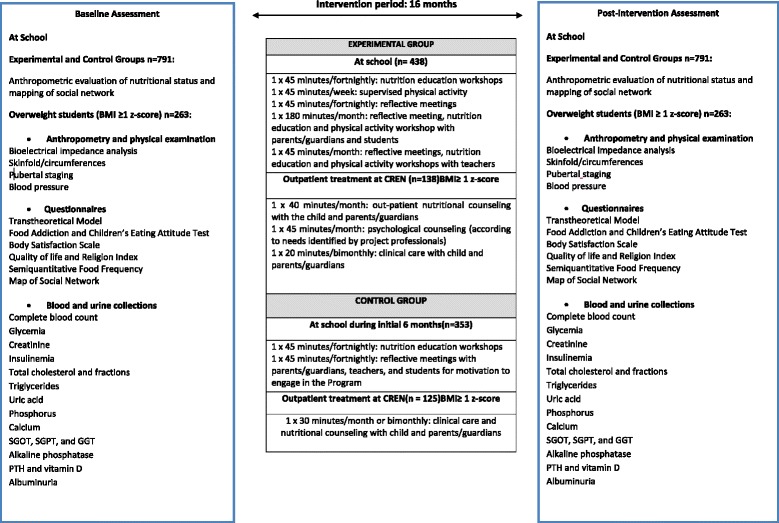
Description of the study and of the Health Multipliers Program Multiplicadores da Saúde (Health Multipliers)

Participants in the experimental group are being enrolled in weekly theoretical/practical group activities at the school throughout the 16-month intervention period. Additionally, students with excess weight receive outpatient care at CREN (Fig. 1).

### At school

All students of the 3rd, 4th, and 5th grades participate in the activities in their respective classrooms (Fig. 1). The intervention groups consist of weekly reflective meetings conducted by the psychologist, nutrition education workshops conducted by two dietitians, and physical activities supervised by the specific physical educator of the project in addition to the routine classes of physical activity included in the school curriculum. Further, the research team holds monthly meetings focusing on nutrition education and improved lifestyle for families and students. The following activities are held during these meetings: nutrition workshops, reflective meetings, and physical exercise. Teachers participate in the entire program as collaborators, favoring the youth participation in the proposed activities. Specific monthly meetings are also being conducted for teachers. Moreover, the project team is available to all teachers who wish to receive individualized nutritional care, offering anthropometric and body composition evaluations and nutrition advice.

### Description of the activities at school

- **Reflective Meeting**
  The reflective meeting is a psycho-educative group activity for seeking solutions and alternatives for common and significant issues of the existence. The theoretical base of this practice is the existential phenomenology and the dialogical group meetings proposed by Paulo Freire [18]. This approach was chosen because the phenomenological exploration in psychology allows a broad understanding of the human experience, with a focus on the meanings, motivations, and decisions for actions. According to *Van Manen & Adams*, it can allow the participants to suddenly see or grasp a human phenomenon in a way that enriches their understanding of everyday life experiences. Such a sudden insight may transform their being and, therefore, their practices [19]. The present protocol employed this methodology as the result of years of experience at CREN regarding treatment in complex environments, such as families facing many difficulties in their relationships. The reflective meeting provides an atmosphere to build new paths for the issues discussed and to explore, by exchanging experiences, better living practices on a daily basis.
  The meetings occur weekly in groups of about 35 students at a time and last 45 min. Examples of the themes discussed are presented in Table 1. Each theme has been designed to meet the demands identified in their daily life. The psychologist coordinates the group activities in cooperation with another professional aid, who records key information during the sessions. This activity is also being conducted with parents/guardians and teachers.
  The meetings are designed in accordance with the structure suggested by Szymanski [18], with the following steps: planning, preparatory activity, reflection focused on the demand, and final synthesis.
- **Nutritional Education Workshop**
  The nutritional education workshop is held in a playful way and includes lectures, videos about childhood obesity and magazine clippings, pictures, posters, slide show, etc. on healthy eating. Examples of the themes discussed are presented in Table 1. The main objectives are to keep students motivated for progressive, healthy changes in eating habits, and nutritional information. This workshop is also being conducted with parents/guardians.
  The workshops occur weekly in groups of 35 students at a time and last 45 min. The meetings are conducted by two experienced and trained dietitians and follow the same dynamics and methodology of the reflective meetings.
- **Supervised Physical Activity**
  Once a week, the physical educator conducts physical activities in an adequate space at the school, with groups of about 35 to 40 children for 45 min. The main objective is to encourage the practice of regular physical activity and to teach the students practices that can be replicated at other times in daily life, focusing on youth autonomy and leadership. To favor protagonism, new rules for the games are discussed and created after debate, with the aim of improving collaboration between teams and partners. Tournaments that involve all classes are also being conducted. The descriptions of the activities are presented in Table 1.
  The meetings with the parents are organized following the reflective meeting methodology and aim to favor the appropriation of physical activity care by all family members, to teach relaxation techniques, and to promote closer ties between parents/guardians and students using the physical activities as a mean. Cooperative games and activities that favor corporal contact between parents/guardians and students are also performed. These activities end with a moment of final reflection to address the apprehension of the meaning of what was accomplished.

**Table 1:** Group activities with students and parents/guardians at school

Themes	Objectives
Reflective Meetings With Students	
Establishing bonds of trust	Introduction of the research team to the students Creating intimacy between research team and students Clarification on the school Program “Health Multipliers”
Creating a personal diary to describe life events, perceptions, likings, worries, desires, etc	Creating an instrument that fosters dialog among students, between students/teachers or research team Teaching the students how to use the diary regularly
Is it possible to change?	Discussing how students conceive themselves in that moment and what they would like to change in their lives Discussing about body self-image Discussing about which changes are possible and how to achieve them Considering obstacles that make changes in a difficult life Sharing experiences of students who have managed to change something in their lives, in particular, considering how they overcame obstacles in the classroom Discussing about how it was possible for them to overcome such obstacles
How do I feel? What do I do?	Favoring self-perception of their feelings at any given moment Encouraging self-perception of more recurring feelings in their lives Understanding how their feelings interfere on their day-to-day actions Discussing if anyone in the group has ever experienced a situation in which, because of being under the influence of a strong feeling, has done something he/she regrets Discussing if anyone, by perceiving feelings, was able to avoid some negative situation Discussing about how did it happen.
How to deal with difficulties?	Remembering situations that caused negative feelings Remembering how they reacted to such situations Remembering situations in which the students have actually succeeded in reacting in a satisfactory manner Discussing about how students find the solution and how they can extend this solution to the lives of others
How to have fun in the real world?	Understanding how students usually have fun in daily life Considering the context in which fun happens: with whom, where, when, etc. Remembering situations where they have been able to enjoy without having to use electronic equipment Discussing about how that student found the solution, and how they can extend this solution to the lives of others
Getting to know each other	Understanding what the students usually do together Considering the context where they meet and interact Understanding the barriers to meet friends Remembering situations where they met to have fun Understanding how they can expand social interaction among them
Reflective Meetings Parents/guardians	
How to find time?	Understanding how they reconcile healthy habits and commitments, such as work, home care, children, and so on Understanding if “health” is a priority in their lives Reflecting on the consequences of a sedentary life Understanding if they consider an active life for their children important, and how they encourage them to do so Reflecting on moments in which it was possible to find time within the routine of each one to practice some activity that promotes their own health as well as their children’s
Dialogue, intimacy, and leadership	Understanding how they engage in dialog with their children Understanding if there is freedom of both parties—children and parents—for dialog Promoting dialog between the parties Understanding how they perceive themselves as protagonists of their own life and family life Reflecting on how to improve dialog between the parties and whether anyone in the group has already succeeded at this
Nutrition Workshops Students	
Knowing the food routine of the students	Understanding their eating routine and promoting reflection on how to improve it
Healthy versus unhealthy foods	Identifying food preference Reflecting on concepts of healthy and unhealthy foods
Knowing the ultra-processed food	Discussing the main nutritional differences between natural, minimally processed and ultraprocessed foods by looking at labels and packages of industrialized products Reflecting on the impact of the consumption of these foods on their health
What do I eat?	Comparing the amounts of sugar, fat, and salt of natural and ultra-processed food.
Olympics in Brazil: what do athletes eat?	Reflecting on feeding habits of famous athletes and comparing them with food choices of students
What food is this?	Knowing and identifying natural foods
Cooking workshop: how to prepare tasty and delicious foods in a healthy way	Learning easy recipes for making ice-cream using real fruits in the preparation Awakening interest in developing these recipes at home
Nutrition Workshops Parents/guardians	
Breakfast and quality of life	Developing easy, inexpensive recipes for a breakfast routine Talking about the relationship between eating breakfast, weight gain and the quality of life
The use of milk	Discussing the consumption of milk from birth to the present moment in their children’s life Knowing their preferences and frequency of milk consumption Remembering the milk recipes developed by the family Making together preparations of milk mixed with fruits Sharing the experience of making and tasting the recipes developed Discussing how to improve the family diet routine by milk consumption
Motivation to achieve nutritional goals	Reflect, through the use of motivational sentences, about the difficulties they face to achieve the nutritional goals propose during the reflective meetings Discussing the concepts that each one has about certain food, body composition, and life habits
Physical activity Students	
The importance of physical activity for health	Raising awareness among students about the importance of regular physical activity for health and well-being maintenance
Stretching and relaxation activities	Guidance on stretching and relaxation techniques so students can apply them in everyday life
Folk dance	Constructing a folkloric dance choreography involving students and teachers, to present to parents and communities during a school festivity
Knowing about sports and competitions	Two sports modalities were chosen, rugby and free dance, after discussion with the students. The students then studied the theory of each one of them, familiarized with the rules and applied the knowledge to propose new rules during the training Physical activities involved exercises of strength, aerobics, balance, and resistance, culminating in a sporting tournament
Competition between classes	A competition between classes occurred at the end of the first semester of intervention, counting on the involvement of children from three different classes and their teachers
Physical Activity (Parents/guardians)	
Dialog with parents	Dialog with parents/guardians about the importance of physical activity to the health and well being of their children
Playing with parents	Developing plays to support contact and interaction between parents/guardians and children
Relaxing with parents	Developing exercises of relaxation involving parents/guardians and their children to support the bond between them

### Outpatient treatment at CREN

All overweight/obese students, Body Mass Index (BMI)-for-age = weight/height in square meters ≥1 Z score, *n* = 138, have been invited to attend outpatient care at CREN with their parents. At the Center, they receive nutritional counseling, clinical care (the frequency is according to the presence/type of comorbidities), and psychological counseling according to the needs identified by the research team and/or family (Fig. 1).

### Description of the outpatient treatment at CREN:

- **Nutritional Counseling**
  At the first meeting, the dietitian identifies the readiness for change in the patient, according to the methodology of the Transtheoretical Model Test (TTM) for Behavioral Change [20]. After classifying the stage of change, the consultation focus is then nutrition counseling using the strategy of Motivational Interviewing (MI) [11]. MI is a client-centric directive counseling that aims to stimulate behavior change, helping participants to explore and resolve their ambivalence (conflict between two paths to follow, in this case, healthy or unhealthy eating habits) [12]. The dietitian, therefore, does not prescribe eating plans or strategies for calorie control, but focuses on the identification of the state of readiness to change, intervene to reinforce the positive steps taken by the patients and their families, and establish new goals by providing new information on healthy lifestyle. These targets are based on the Guidelines for the Treatment of Childhood Obesity [10, 14, 21] as well as the recommendations of the World Health Organization(WHO) [2] and the Brazilian Food Guide(BFG) [22]. The aims of the strategy are as follows: reducing the consumption of sugary drinks with progressive increase in the intake of drinking water, reducing the consumption of fried and ultra-processed of foods by replacing them with options of raw or minimally processed foods, increasing in the consumption of fruits and vegetables (target of 3 to 5 servings/day), avoiding sedentary lifestyle (decrease screen time to less than 2 h/day), and increasing regular physical activity (60 min goal a day/5 days a week). These targets are reassessed every new visit and redefined or strengthened for the following period.
  In addition, a 24-h dietary recall is performed during the consultation, as a tool to guide the advice and to setting monthly goals for the patient. The duration of this consultation is 40 min (first visit) and 30 min (return visits).
- **Clinical care**
  The clinical evaluation is based on the overall assessment of the patient, including physical examination to define the pubertal stage and blood pressure, and evaluation of laboratory parameters. Participants with comorbidities, as for example, diabetes, dyslipidemias, hypertension, insulin resistance, hyperinsulinemia, or hyperglycemia are treated according to the Guidelines of the Brazilian Society of Pediatrics [23]. Participants with orthopedic, dermatological, renal, hepatic, respiratory, or psychological alterations are referred to the appropriate specialists.
  Patients diagnosed with deficiency/insufficiency of vitamin D (25OHD <20 ng/ml and <30 ng/ml, respectively) are treated with proper supplementation [24]. Participants of the experimental group receive the supplement at school with a dosage of 50,000 IU of cholecalciferol once a week for 6 weeks, receiving gelatinous capsules administered under the supervision of the research team. After the supplementation period (between 30 to 60 days after the end of treatment), serum 25OHD is checked. Participants with serum 25OHD concentrations of ≥30 ng/ml are provided cholecalciferol supplement (D3) of 600 IU/day for maintenance for three consecutive months. Participants who do not achieve recovery of vitamin D deficiency after this period receive further supplementation of 50,000 IU of cholecalciferol, once a week for other 6 weeks, followed by repeat evaluation of D 25OH after this period.
- **Psychological Counseling**
  Students of the experimental group identified with some significant psychological distress during nutritional or medical appointments and even those actively seeking help are met individually through psychological counseling sessions. In these sessions, the therapist searches jointly with the patients an understanding of their demand and life problem, as well as a possible way to solve it [25].

#### Control group

##### 2.9.1.1.At school

The students in the control group (*n* = 353) participate in weekly meetings with the whole class for the first six months, with the purpose of motivation for adopting a healthy lifestyle (Fig. 1). All students of the 3rd, 4th, and 5th grades participate in these activities. Weekly reflective meetings were conducted by the psychologist, as well as nutrition education workshops led by the two dietitians. In addition, three meetings are held with parents and teachers where the following activities are performed: the importance of having a healthy lifestyle, eating breakfast, and teaching healthy recipes. After this motivation period, all overweight students were referred for outpatient treatment at CREN.

### Outpatient treatment at CREN

Overweight/obese students of the control group (BMI ≥ 1 Z score, *n* = 125) were referred for outpatient care in accordance to the routine offered by CREN. In this case, the frequency of visits varies according to the severity of nutritional diagnosis (monthly or bimonthly). They also receive pediatric care (frequency according to the presence of comorbidities), psychological counseling by medical referral, and may voluntarily participate in recreational tours or attend groups for physical activity (Fig. 1).

Patients of the control group receive the same prescription of cholecalciferol described for the experimental group, at the same dosage and for the same treatment period. The difference in this group is that parents/guardians are asked to assume the task of taking care of the supplementation at their homes.

#### Data collection

##### 2.10.1.1.Weight and height

Weight was measured using a digital portable scale (Plenna® brand MEA 07400, São Paulo, Brazil) with a maximum capacity of 200 kg and a precision of 50 g. Participants were weighed using light clothing, without shoes and accessories. Height was measured using a portable stadiometer for field research, with a precision of 1 mm. (Height Exata®, São Paulo, Brazil), with subjects in the vertical position, wearing light clothes, without head garments, undone hairstyle, and head positioned in the Frankfurt plane.

The nutritional status of the students was evaluated by calculating the Z score of the BMI -for-age, using WHO AnthroPlus (version 1.0.4, 2009), at the beginning and at the end of the intervention period (Fig. 1).

##### 2.10.1.2.Mapping of student social network

A map of the social network of the students is being designed according to the methodology described by Sanicola [26], at the beginning and at the end of the intervention period (Fig. 1). This technique used in social sciences allows the mapping of the qualitative (type) and quantitative (number) relationships that make up the relational web of an individual. The map is composed of the primary (friends and family) and secondary (institutions) networks.

People in the primary network groups are united by ties of kinship, neighborhood, and friendship that influence, in some way, the individual’s life condition and choices [27]. It is known that relationships between friends are extremely important for the profile of consumption. The secondary network maps the relationship of the individual to institutions, such as schools, churches, hospitals, among others [26].

The map is developed in the classroom after an extensive explanation and under the supervision of the researcher in charge of the activity. Students are asked to paste on a white sheet colored circles (green = relatives, blue = friends, yellow = neighbors) that represents the number of their relationships. The number of circles and the relationship types are then quantified. The school is considered the secondary network of reference. Students are also asked to write in an ellipse marked in the same white paper the number of colleagues/teachers/employees who they considered friends or with whom they have a personal relationship at the school environment.

### Outcome measures

The nutritional profile of all students will be compared at the beginning and end of the school intervention. The incidence of overweight/obesity will be calculated according to the percentage of participants who were not overweight/obese at the start of the intervention but may be overweight/obese by the end of the intervention period. The prevalence of overweight/obesity will be defined as the number of individuals who remained overweight/obese during the study, and remission will be calculated according to the number of participants who are no longer overweight/obese at the end of the study. In addition, the social network and its evolution over the intervention will be mapped and compared to the nutritional profile.

#### Data collection of students with excess of weight (*n* = 263)

Data collection of students with excess of weight (overweight/obese) from experimental and control groups occurred at the beginning of the study and will be repeated 16 months after the initiation of intervention. Figure 1 contains the information that is being collected. Additionally, Table 2 describes the methods used to evaluate biochemical parameters.

**Table 2:** Biochemical evaluation

Test	Technique/Machine/Manufacturer	Measures
Complete BloodCount	CITOQUÍMICO/ISOVOLUMÉTRICO/	Variável de acordo com cada componente do exame
Glutamicoxalacetic transaminase(SGO-T)	CINÉTICO–IFCC/AU5800BeckmanCoulter /	U/L
Glutamic-pyruvictransaminase(SGP-T)	CINÉTICO–IFCCWAU5800/BeckmanCoulter	U/L
Gamma-Glutamyltransferase(GGT)	CINÉTICO–SZASZ/AU5800/BeckmanCoulter	U/L
Total cholesterolandfractions	COLORIMETRICO-PEROXIDASE /AU5800/BeckmanCoulter	mg/dl
HDL	COLORIMETRICO–ENZIMÁTICA/AU5800/BeckmanCoulter	mg/dl
VLDL	EQUAÇÃO DE FRIEDWALD/AU5800/ Beckman Coulter	mg/dl
LDL	EQUAÇÃO DE FRIEDWALDAU5800/Beckman Coulter	mg/dl
Calcium	COLORIMETRICO/AU5800/BeckmanCoulter	mg/dl
Phosphor	COLORIMETRICO/AU5800/BeckmanCoulter	mg/dl
Alkalinephosphatase	AMP IFCC/AU5800/Beckman Coulter	U/L
Fastingblood glucose	COLORIMETRICO ENZIMÁTICO/AU5800	mg/dl
PTH	QUIMIOLUMINESCÊNCIA/Architect/Abbott	pg/ml
Insulin	QUIMIOLUMINESCÊNCIA/Architect/Abbott	U/L
Uric acid	ENZIMATICO–URICASE/AU5800/BeckmanCoulter	mg/dl
Creatinine	COLORIMETRICO–JAFFE/AU5800/Beckman Coulter	mg/dl
Albuminuria (isolated sample)	IMUNOTURBIDIMETRIA/AU5800/BeckmanCoulter	mg/g/creatinina na urina
Vitamin D3–25 hidroxicholecalciferol	QUIMIOLUMINESCÊNCIA/Architect/Abbott	ng/ml

### Circumferences and body composition

Measurements of the circumference of the neck, waist, hip, and calf are performed using an inelastic fiberglass band (Sanny®, American Medical do Brasil Ltda) with accuracy of 1 mm. The circumference of the neck is measured below the laryngeal prominence, perpendicular to the largest axis of the neck. The waist circumference is measured by the diameter at the midpoint between the last costal border and the iliac crest [28]. To evaluate abdominal fat, values above the 80th percentile (P80) will be considered as accumulation of abdominal fat (Taylor et al. 2000). Hip circumference is measured at the level of maximal extension of the gluteus [29]. All circumference measurements are repeated twice and registered immediately after reading the measurements. Tricipital, subscapular, and calf skinfolds are measured using a skinfold caliper (Sanny®, American Medical do Brasil Ltda) with 0.5 mm accuracy according to Lohman recommendations [30]. Both triceps and subscapular skinfolds are measured with the students standing erect and the arms naturally hanging. All skinfolds are measured on the right side, in triplicate.

The percentage of body fat (% GC) will be estimated from the sum of triceps, subscapular, tricipital, and calf skinfolds, using the predictive equations proposed by Slaughter et al. 1988, and Hoffman et al. 2012 for Brazilian students [31, 32]; and through Bioelectrical impedance (Biodynamic Body Composition Analyzer, model 310, version 8.01- Biodynamics Corporation, Seattle, USA). This evaluation is performed with the student lying for five to 10 min in the supine position on an insulated mat of electric conductors, without carrying any metallic object, with the legs apart, hands open, according to the methodology described by Lukaski et al. [33]. Students and their caregivers receive prior guidance for proper preparation in the hours prior to the test, i.e., avoiding consumption of alcohol and caffeine (coffee, tea, chocolate) 24 h before the test, not engaging in intense physical activity, avoiding any heavy meal 4 h before the test, and urinating about 30 min before the test.

### Blood pressure

Blood pressure is measured using an oscillometric device (OMROM-HEM-Healtcare 7113® Intellisense –OMRON, DALIAN CO, LTD-CHINA). The measurement is done on the right arm, supported at the level of the heart. Three measurements are taken with a 2-min interval between them, with the student previously relaxed sitting with his back on the chair and feet resting on the floor in an isolated room. Blood pressure will be classified according to the mean of the measurements; analyzed in percentiles, according to age, gender and height; and compared to the values published in the National High Blood Pressure Education Program, 2004 [34].

### Pubertal staging

The evaluation of the pubertal stage is made by the student’s self-evaluation method from a table with images of the five pubertal stages of Tanner, according to gender [35]. For girls, the board is presented with staging for breasts (M) and pubic hair (P). For boys, the plank contains of staging of the external genitalia (G) and pubic hair (P). The evaluation is done individually and the student is asked to point out his stage according to his perception. This evaluation is carried out in a quiet and reserved environment, solely in the presence of the evaluator.

### Evaluation of the quality of life and religiosity

The quality of life is evaluated through the Pediatric Quality of Life Inventory questionnaire - PedsQL ™ 4.0 (ages 8–12 years) validated to Portuguese [36, 37]. For its application, a properly trained researcher invites each student individually to a quiet environment to answer the questionnaire at school. The researcher reads the questions and takes personal notes after an oral response of the student. This methodology was used as this questionnaire contains some questions that are difficult for students with poor schooling abilities to understand. The questionnaire has four areas: (a) health evaluation by assessing the difficulties that the student encounters when performing activities such as running, walking, bathing, carrying something heavy, pain or lack of energy; (b) problems with feelings (scared, sad, angry, sleeping difficulties, worries about the future); (c) problems about how to get along with others; and (d) problems about performance at school.

A growing number of studies have shown that religiosity, although it does not have a direct action on physical health, influences strongly psychological well-being and adherence to treatments. For this reason, the student’s religious practice is being investigated. The results will be evaluated in relation to adherence to the proposed protocol and performance during treatment. A religious scale developed by researchers from the American University of Duke, the Duke Religious Index (DUREL), validated for the Brazilian population, is being used [38, 39]. This questionnaire is being applied by a properly trained interviewer in a quiet environment. The interviewer reads the questions, explains the meaning of the words to the student if necessary, and registers the answers him/herself. The instrument accesses the three major dimensions of religiosity: organizational and non-organizational religious activities and intrinsic (subjective) religiosity [39].

### Evaluation of food addiction and eating attitudes

The Children’s Eating Attitudes Test (ChEAT) validated to Portuguese for this age group is used for the evaluation of abnormal eating attitudes and behaviors [40, 41]. The questionnaire has three domains: (a) food preoccupation, (b) restrictive and purgative behaviors and (c) oral control. A trained dietitian is responsible for applying the questionnaire at school. The questionnaire is applied in two students at the same time. The students are separated so that they are not able to see one another and are instructed not to read their answers aloud. Each application lasts about 45 min. The students receive the questionnaires and a pen to mark the response. The lines are alternated with white and gray lines to make it easier for the student to follow the line and not mark the answer to the wrong question. The researcher reads each question, while the students follow the reading in their own questionnaires. To ensure the quality of the application and avoid bias through tiredness and repetition, up to 10 students are invited to answer the questionnaires each day. This information will aid the dietitian in charge of the outpatient consultations to assist the student in behavior change and to create strategies that can help the nutritional recovery process.

The protocol also includes the use of the Yale Food Addiction Scale (YFAS) [42] that is applied in exactly the same way as the Children’s Eating Attitude Test. This scale has not been validated in Brazilian children and the researchers extended the translation to Portuguese. The results will be compared with all the other information collected in this intervention protocol.

### Transtheoretical model test

The readiness for change of the students is being evaluated according to the Transtheoretical Model test. This test addresses five stages of behavior: pre-contemplation, contemplation, preparation, action, and maintenance [20, 43]. The main characteristic of the pre-contemplation stage is the lack of willingness to change. This stage is characterized by a resistance to acknowledge the error and to modify it. Those in the contemplation stage are aware of their problem and are seriously considering changing the behavior but have not yet taken any initiative, do not know where to start, or do not feel prepared to change. In the preparation stage, the subject wants to change behavior in near future, claims to be motivated, and sees that change may occur in the following months. The action stage involves a previous occurrence of a behavioral and/or environmental change. Subjects at this stage underwent real experiences to stop the problem and have overcome previously perceived barriers. The maintenance stage occurs when the subjects have changed their behavior for more than six months, and the intervention aims to maintain the habits acquired. The questionnaires with the food behavior and physical activity algorithms are applied exactly in the same way as the Eating Attitude Test described before. This information will aid the dietitian during the outpatient consultations.

### Figure rating scale

Body image is closely linked to the development of eating disorders and quality of life and is a determining factor for readiness to change food behavior. The usual method of evaluation is the scale of silhouettes ranging from the leaner to the more obese [44]. The scale has several advantages, since it is easy to apply (it can be self-applied), it does not require a great diversity of verbal fluency and it is an inexpensive and didactic instrument, which makes it particularly suitable for the evaluation of the perception of the corporal image, mainly of children [44]. In the case of children, the great concern is the suitability of the scale figures for children’s silhouettes from other countries; therefore, a Brazilian silhouettes scale was used for children from 7 to 12 years of age. The scale was applied at the same time as the other questionnaires at school.

### Food consumption

A Semiquantitative Food Frequency Questionnaire (SFFQ) was used for the estimation of long-term dietary intake [45]. This method also allows the estimation of the amounts ingested per unit of time (day, week, or month). Energy and nutrients are calculated according to the frequency of consumption [45, 46].

The Brazilian SFFQ for adolescents was developed and validated in Rio de Janeiro in 2010 [45]. The questionnaire was pre-tested with 10 adolescents (written informed consent, was obtained from these participants and their parents/legal guardians) living in the same region of the present study, in order to evaluate if the foods that make up the list were included in the habitual repertoire of the population of São Paulo. Simultaneously, we also analyzed the time required for the application of the questionnaire and the receptivity of the adolescents in relation to the research protocol. Before application, the questionnaire was evaluated by the team of dietitians at CREN and was modified and adapted to account for the differences in regional nomenclatures, with inclusion and exclusion of very infrequent food items.

The computer was chosen for data collection. The same interviewer applied the questionnaire individually. A Photo Manual was used to assist recording the portion sizes [46]. To avoid bias due to fatigue and repetition, SFFQ was applied in a maximum of 8 students per four-hour period. Data from the food records will be converted to energy and nutrients using the Nutrition Data System for Research (NDS-R version 2012, University of Minnesota) software.

### Socioeconomic data collection

The collection of information on the socioeconomic profile of the student is performed at CREN during outpatient visits, in order to gain an insight into the student’s family conditions and the environment at their place of residence. Information on the student’s family composition, parents’ level of education, and family income are also registered.

### Outcomes

Recovery of BMI (BMI < 1) or a persistent and progressive reduction in BMI throughout the intervention period will be considered as the primary outcome.

Furthermore, the following are the secondary outcomes expected: reduction of waist and neck circumferences, reduction in body fat percentage, reduction in blood pressure, and improvement of the biochemical profile. The following improvements in social variables are also expected: in the proportion of overweight students motivated to maintain healthy eating habits and in achieving the stablished goals, in the proportion of those motivated to maintain physical activity recommendations, on the average quality of life index, in the proportion of students with a positive body image, recovery of the symptoms of food addiction, and the social network communications regarding excess weight.

### Exclusion criteria

Overweight students of both control and experimental groups were excluded from the data collection if they were reported to have cognitive delay, as per their parents and/or teachers, which could limit their involvement in activities such as answering questionnaires. In addition, were excluded those with motor limitations, twins, those taking medications known to affect body weight management, and those with any known family issues that could affect the overall compliance and participation in the Program.

### Analysis plan and statistics

The data will be tabulated with double input. Descriptive statistics will be used to correlate all variables, by using mixed analysis models. Adjustments will be made for age, gender, pubertal stages using IBM Statistical Package for Social Sciences (SPSS Statistics Desktop) base 22.0.

Changes in the outcomes will be evaluated using a fixed-effects model, and covariate adjustment will be performed to account for the difference in sizes between both groups. Missing data will be identified in each subgroup. An imputation model based on the Multiple Imputations by Chained Equations (MICE) method will be applied (package MICE 2.25, running on R version 3.3.2) [47]. The imputation model will include all the baseline features in addition to age, gender, and classroom. According to this method, a suitable number of imputation models to account for a 95% CI is 59 (we will assume a worst-case scenario and use the maximum missing proportion). Regarding the number of iterations, as suggested in [48], we will use 5 iterations and the Predictive Mean Matching (PMM) method for every outcome variable. After generating imputed models, they will be pooled according to Rubin’s rule in order to obtain valid statistical inferences [49].

Changes in remission, incidence, and prevalence of obesity in the experimental group and control will be evaluated at the end of the study. Odds ratios adjusted for age, gender, and other baseline measures will also be calculated.

## Discussion

The progressive increase in the prevalence of overweight/obesity requires the implementation of effective interventions that influence the pandemic growth of this disease and its control have scientific and social relevance, especially in low-income populations. The complexity of the factors that involve the treatment and prevention of the excess weight makes it challenging to design interventions and test different treatment approaches in different contexts. However, an effective and sustainable protocol for the management of excess of weight, particularly in low-income settings, has not yet been identified in the scientific community.

Many attempts have been made to adequately treat excess weight in childhood and adolescence, but to date, the scientific literature has not been able to answer whether it is possible to recover excess weight in this age group. Moreover, interventions based on the motivation to implement healthy eating habits and lifestyle, as the design adopted in this Program, are new in the literature.

This study provides a multi-component approach to long-term educational and environmental interventions involving the school and family environment, with a focus on the sustainability of the results obtained. This approach can greatly influence the quality of life of the families involved and in the school environment, promoting a reduction in the sedentary lifestyle and consumption of foods classified as non-satisfactory, increasing the consumption of healthy foods, and favoring the construction of a school environment that works as a promoter of student’s health. Therefore, this program may have an impact on the prevalence of excess of weight, and its repercussions may contribute to definitions of public policies at school and community level, as well as provide valuable information for other research groups about the best treatment for excess weight in this age group.

## Abbreviations

BFG: Brazilian Food Guide
BMI: Body Mass Index
ChEAT: Children’s Eating Attitudes Test
CREN: Center for Recovery and Nutritional Education
DUREL: Duke Religious Index
FAPESP: São Paulo State Research Foundation
MI: Motivational Interviewing
MICE: Multiple Imputations by Chained Equations
NDS-R: Nutrition Data System for Research
PedsQL ™ 4.0: Pediatric Quality of Life Inventory questionnaire version 4.0
PMM: Predictive Mean Matching
RECHB: Research Ethics Committee of Human Beings
RTC: Randomized Control Trials
SFFQ: Semiquantitative Food Frequency Questionnaire
SPSS: Statistical Package for Social Sciences
TTM: Transtheoretical Model Test
UNIFESP: Federal University of São Paulo.
WHO: World Health Organization
YFAS: Yale Food Addiction Scale
